# Influence of alcoholism and cholesterol on TSPO binding in brain: PET [^11^C]PBR28 studies in humans and rodents

**DOI:** 10.1038/s41386-018-0085-x

**Published:** 2018-05-03

**Authors:** Sung Won Kim, Corinde E. Wiers, Ryan Tyler, Ehsan Shokri-Kojori, Yeon Joo Jang, Amna Zehra, Clara Freeman, Veronica Ramirez, Elsa Lindgren, Gregg Miller, Elizabeth A. Cabrera, Tyler Stodden, Min Guo, Şükrü B. Demiral, Nancy Diazgranados, Luke Park, Jeih-San Liow, Victor Pike, Cheryl Morse, Leandro F. Vendruscolo, Robert B. Innis, George F. Koob, Dardo Tomasi, Gene-Jack Wang, Nora D. Volkow

**Affiliations:** 1 National Institute on Alcohol Abuse and Alcoholism, NIH, Bethesda, MD 20892 USA; 2 Molecular Imaging Branch, National Institute of Mental Health, NIH, Bethesda, MD 20892 USA; 3 National Institute on Drug Abuse, NIH, Baltimore, MD 21224 USA

## Abstract

Neuroinflammation appears to contribute to neurotoxicity observed with heavy alcohol consumption. To assess whether chronic alcohol results in neuroinflammation we used PET and [^11^C]PBR28, a ligand that binds to the 18-kDa translocator protein (TSPO), to compare participants with an alcohol use disorder (AUD: *n* = 19) with healthy controls (HC: *n* = 17), and alcohol-dependent (*n* = 9) with -nondependent rats (*n* = 10). Because TSPO is implicated in cholesterol’s transport for steroidogenesis, we investigated whether plasma cholesterol levels influenced [^11^C]PBR28 binding. [^11^C]PBR28 binding did not differ between AUD and HC. However, when separating by *TSPO* genotype rs6971, we showed that medium-affinity binders AUD participants showed lower [^11^C]PBR28 binding than HC in regions of interest (whole brain, gray and white matter, hippocampus, and thalamus), but no group differences were observed in high-affinity binders. Cholesterol levels inversely correlated with brain [^11^C]PBR28 binding in combined groups, due to a correlation in AUD participants. In rodents, we observed no differences in brain [^11^C]PBR28 uptake between alcohol-dependent and -nondependent rats. These findings, which are consistent with two previous [^11^C]PBR28 PET studies, may indicate lower activation of microglia in AUD, whereas failure to observe alcohol effects in the rodent model indicate that species differences do not explain the discrepancy with prior rodent autoradiographic studies reporting increases in TSPO binding with chronic alcohol. However, reduced binding in AUD participants could also reflect competition from endogenous TSPO ligands such as cholesterol; and since the rs6971 polymorphism affects the cholesterol-binding domain of TSPO this could explain why differences were observed only in medium-affinity binders.

## Introduction

Alcohol use disorder (AUD) is a chronic relapsing disorder characterized by the inability to stop drinking despite one’s awareness of negative consequences. Repeated exposure to high doses of alcohol has been associated with neurotoxicity that can result in cognitive deficits [1]. There is increased recognition that alcohol-induced neuroinflammation contributes to its neurotoxicity [2]. However, the mechanisms underlying the neurotoxicity from high doses of alcohol are poorly understood.

Microglia (about 10–15% of the brain’s cells) are the resident innate immune cells in the brain and are activated in response to cellular stressors [3, 4]. Once activated, microglia release pro-inflammatory cytokines and chemokines, glutamate, adenosine triphosphate (ATP), and reactive oxygen species [5, 6], all of which contribute to the inflammatory process. In animal models, alcohol can activate microglia and induce the production of inflammatory mediators [7]. Administration of alcohol at doses that mimic binge drinking in humans was shown to activate microglia and increase the release of pro-inflammatory cytokines and chemokines in rodents [2, 8]. 18-kDa translocator protein (TSPO) is expressed in active microglia and is considered to be a marker of neuroinflammation [9, 10] (although TSPO is also expressed in resting microglia, astrocytes, and neurons [11]). A preclinical autoradiography study with the neuroinflammation ligand [^3^H]PK-11195 that binds to TSPO, found that 4 days of binge drinking in rats increased [^3^H]PK-11195 binding in hippocampus and entorhinal cortex [12]. Furthermore, knockdown of TSPO in neurons of male adult drosophilae increased their sensitivity to alcohol’s sedative effects and blocked tolerance development to repeated alcohol exposures, identifying TSPO as a modulator of alcohol’s effects [13].

In humans, alcohol abuse induces inflammation in the brain and body [2], affecting immunity and increasing susceptibility to certain infectious diseases [14]. Moreover, there is evidence that alcohol abuse increases systemic markers of inflammation such as C-reactive protein and cytokines [15]. Similarly, studies using postmortem brain tissue from patients with AUD showed increases in cytokines (monocyte chemoattractant protein 1 [MCP-1]) and in markers of microglial activation [2, 16]. Gene expression studies on postmortem brains have also reported increases in genes involved with inflammation in AUD [17]. In contrast, brain imaging studies using PET and the TSPO ligand [^11^C]PBR28 have reported decreased binding in AUD participants compared to controls [18, 19]. One study showed a 10% reduction in PBR28 binding in the brain of 15 subjects with moderate AUD compared to 15 controls [18], although this effect was no longer significant with the removal of the one patient with 24 days of abstinence. Another study found a 20% reduction in PBR28 binding only in the hippocampus in 9 AUD subjects compared to 20 controls that was driven by 3 AUD high-affinity binders (*TSPO* rs6971 homozygous), whereas there were no group differences in medium-affinity binders (rs6971 heterozygous) [19]. Although both studies seem to indicate decreases in PBR28 binding in AUD, the effect sizes are small and restricted to some of the AUD patients, but not others.

Here, we further characterize the effects of heavy alcohol exposure on neuroinflammation as assessed with PET and [^11^C]PBR28. In order to control for heterogeneity among AUD patients, we conducted parallel studies in humans and in a rodent model of alcohol dependence. Based on animal findings with the TSPO ligand [^11^C]PK-11195 [12], we initially hypothesized higher [^11^C]PBR28 binding in AUD patients and in alcohol-dependent compared to -nondependent rats, reflecting neuroinflammation after chronic alcohol consumption. However, given the two recent studies showing the opposite to the expected finding of lower [^11^C]PBR28 binding in AUD participants versus controls [18, 19], we expected to replicate these findings in our clinical and preclinical data set. Additionally, TSPO has a cholesterol-binding domain in its fifth transmembrane loop and cholesterol binding leads to structural changes in TSPO shifting the equilibrium toward the translocator monomer [20]. Because alcohol can modify plasma cholesterol levels [21, 22], we also explored the association between cholesterol levels and [^11^C]PBR28 binding in humans and hypothesized an inverse relation between them. Finally, because *TSPO* deletion mutations in rodents and the rs6971 *TSPO* polymorphism in humans alters adrenocorticotropic hormone (ACTH)-induced plasma cortisol concentrations [23], we also explored the relationship between plasma ACTH and cortisol levels and whole brain [^11^C]PBR28 binding.

## Materials and methods

### Human study

#### Participants

Nineteen patients with AUD and 17 HC completed the study. Groups were matched for age, gender, body mass index (BMI), and TSPO genotype (see Table 1 for demographics and clinical characteristics). Participants were medically screened to exclude ferromagnetic implants, major medical problems, chronic use of psychoactive medications, neurological problems, or head trauma; and current diagnosis of a substance use disorder (other than alcohol abuse and/or dependence in the AUD group or nicotine dependence for either group), past history of drug abuse or other psychiatric disorders that needed treatment as assessed by the Structured Clinical Interview for the Diagnostic and Statistical Manual of Mental Disorders (DSM-IV) [24]. Women were studied in the mid-follicular phase and were neither pregnant nor breastfeeding. AUD participants were scanned after 2.8 days of abstinence (range, 0–7 days) and had at least 5 years’ history of heavy drinking. All participants were free of psychoactive medications within 24 h of study procedures (except benzodiazepines for detoxification in the AUD group; *n* = 5), and had a negative urine drug screen on days of testing. *TSPO* single-nucleotide polymorphism (SNP) rs6971 was determined on the day of screening, as described in ref. [25], and low-affinity binders of [^11^C]PBR28 to TSPO were excluded. Participants provided written informed consent to participate in the study, which was approved by the Institutional Review Board at the National Institutes of Health (Combined Neurosciences White Panel). Participants were scanned between June 2015 and April 2017. On the day of screening, participants completed the timeline followback (TLFB) to assess daily alcohol consumption in the 90 days prior to the study [26], the lifetime drinking history (LDH) to assess lifetime alcohol consumption, the alcohol dependence scale (ADS) to assess severity of dependence [27], the Wechsler abbreviated scale of intelligence (WASI-II) subtests matrix reasoning and vocabulary as a proxy for general intelligence [28], and the state–trait anxiety inventory (STAI) [29]. Plasma cholesterol, cortisol, and ACTH levels were acquired using standard NIH Laboratory of Medicine procedures, and blood labs were drawn early morning (between 8 a.m. and 11 a.m.) while participants were fasting.

**Table 1:** Demographics and clinical characteristics of study participants

	**AUD (*n*** = 19)		**HC (*n*** = 17)		
**Characteristic**	**Mean**	**SD**	**Mean**	**SD**	***p*** **value**
Age, years	47.6	10.1	47.5	10.9	0.9
BMI	26.6	4.2	27.8	3.3	0.3
Gender	5 female		8 female		0.3
WASI full IQ score	89.9	16.5	103.2	18.4	**0.03**
*rs6971* genotype	11 high		11 high		0.7
	8 medium		6 medium		
Smoking	10 smokers		0 smokers		**<0.0001**
TLFB average drinks/day	8.9	4.9	0.1	0.2	**<0.0001**
TLFB drinking days/week	5.9	1.5	0.4	0.7	**<0.0001**
Alcohol drinking years	29.5	13.5	16.2	17.6	**0.015**
LDH (kg)	1439	1331	34	494	**<0.0001**
Abstinence (days)	2.8	2.4	4081	1053	**0.01**
ADS	13.5	7.6	0.1	0.3	**<0.0001**
STAI trait	38.9	11.8	26.7	6.2	**0.001**

*P* values in bold are considered statistically significant

*ADS* addiction severity index, *AUD* alcohol use disorder, *BMI* body mass index, *HC* healthy controls, *LDH* lifetime drinking history, *STAI* state–trait anxiety inventory, *TSPO* translocator protein

#### PET study and image data acquisition

All participants underwent brain imaging with [^11^C]PBR28 PET and MRI. [^11^C]PBR28 was produced as described by Fujita et al. [30]. Mean (±SD) injection dose (ID) of [^11^C]PBR28 was 18.8 ± 0.7 mCi (range, 15.5–19.4 mCi), which was injected intravenously over a 1 min period. Mean molar activity (A_m_) of [^11^C]PBR28 at the time of injection did not differ between groups (mean AUD = 2.4 ± 1.1 Ci/μmol, mean HC = 2.9 ± 1.5Ci/μmol, *p* > 0.05) at the time of injection. Prior to tracer injection, a transmission scan was obtained using cesium−137 to correct for attenuation.

Dynamic [^11^C]PBR28 PET scans were performed in list mode using a high resolution research tomograph (HRRT; *n* = 24) (Siemens, Knoxville, TN, USA) or a GE Advance (*n* = 12) (GE Healthcare, Waukesha, WI, USA). There were no effects of scanner type on whole brain [^11^C]PBR28 binding in the overall sample (ANCOVA corrected for genotype: *F*_33_ = 1.1, *p* = 0.3), and scanner type did not differ between groups (*χ*^*2*^ = 0.22, *p* = 0.6). The time of injection ranged from 10:03 a.m. to 2:34 p.m. There were no group differences in injection time (*t*_34_ = 1.4, *p* = 0.2) and no effect of injection time on whole brain [^11^C]PBR28 binding in the overall sample (Pearson’s correlation corrected for genotype *r* = 0.1, *p* = 0.5, ns). We therefore did not include scanner type or injection time as covariates in our analyses. [^11^C]PBR28 PET raw data were reconstructed with a 3D-ordered subset expectation maximization (OSEM) algorithm to generate 27 frames of data for each subject. During the 90-min scan session, 23 time points of arterial blood samples were collected from the radial artery. Both whole blood and the corresponding plasma samples were counted and 16 plasma samples were analyzed with radio high-performance liquid chromatography (HPLC) to quantify the fraction of intact [^11^C]PBR28 and the free fraction (*f*_P_) of [^11^C]PBR28 in plasma [30]. There were no differences in plasma protein binding (*f*_P_) between AUD and HC (mean AUD = 0.016 ± 0.005, mean HC = 0.018 ± 0.004, *t* = 0.88, *p* = 0.4).

#### MRI acquisition

For structural magnetic resonance imaging (MRI), T1-weighted 3D magnetization prepared rapid acquisition gradient echo (MPRAGE; TR/TE = 2200/4.25 ms, 1-mm isotropic resolution) pulse sequences was acquired with a 3T Prisma Scanner (Siemens Medical Systems) [31] to provide anatomical coregistration for [^11^C]PBR28 images and to control for potential brain atrophy in AUD participants.

#### Image preprocessing and regions of interest

Analysis of functional neuroimages (AFNI) and functional software library (FSL) tools were used for spatial normalization and coregistration to the Montreal Neurological Institute (MNI) space [31]. Briefly, T1-weighted MR images were aligned along the AC-PC line. Average [^11^C]PBR28 images were coregistered to the realigned MRI images. The corresponding spatial transformation was applied into the [^11^C]PBR28 dynamic images. The FreeSurfer image analysis suite (v 5.3.0; http://surfer.nmr.mgh.harvard.edu/) was used to delineate regions of interest (ROIs; whole brain, cortical gray matter, white matter, hippocampus, and thalamus) in the subject’s anatomical space to generate time–activity curves for [^11^C]PBR28 within these ROIs.

#### [^11^C]PBR28

Standardized uptake value (SUV) calculated by body weight and injection dose for each subject was used for normalization of both blood input function and image data. A three-exponential function and a sigmoidal function were used to build up the models of plasma and parent [^11^C]PBR28 fraction, respectively. The PMOD-PKIN tool (v 3.8 PMOD Technologies, Zurich, Switzerland) was used to generate the total distribution volume (*V*_T_) using a 2-tissue compartment model (2TCM), based on time–activity curves of the ROIs and the blood input function model. Spatially normalized dynamic [^11^C]PBR28 images were transformed to *V*_T_ parametric images using Logan analysis with the PXMOD tool (v 3.8) (PMOD Technologies Ltd., Zurich, Switzerland).

#### Statistical analysis

Group comparisons on the ROI measures were performed with SPSS version 20. Multivariate analyses were performed with group (AUD/HC) and genotype (rs6971 heterozygous or medium-affinity binders and homozygous or high-affinity binders) as between-group variables, and ROIs as the dependent variables, using Roy’s largest root, and age as a covariate. Effect sizes are reported as partial eta-squared (*η*^*2*^). Post hoc multivariate and univariate tests on differences between AUD and HC were performed for medium-affinity and high-affinity binders separately. We tested associations between whole brain [^11^C]PBR28 and plasma cholesterol, cortisol, and ACTH. Additionally, group differences in gray matter volume (GMV) were explored using voxel-based morphometry (Supplemental Material 1), which revealed decreased GMV only in the bilateral temporal cortex in AUD. The average of responses to obtain alcohol performed by dependent and nondependent rats was compared with an unpaired Student’s *t* test. The results were reported as mean ± standard error of the mean. Significance threshold for all tests was set at *p* < 0.05.

Correlational analyses between whole brain [^11^C]PBR28 *V*_T_ and cholesterol, cortisol, and ACTH were performed in groups combined, and separately for AUD and HC, using partial correlations in SPSS 22, with rs6971 as covariate. We also explored Pearson’s correlations for each genotype separately.

### Rodent study

#### Rats

Adult male Wistar rats (*n* = 20) were purchased from Charles River (Kingston, New York, USA) and weighed 250–300 g at the beginning of the study. Rats were group housed (2–3 per cage) in standard plastic cages lined with woodchip bedding and maintained under a reverse 12 h/12 h light/dark cycle (lights on at 8 p.m.) at 21 ± 2 °C with ad libitum access to food and water. All procedures were conducted according to the National Institutes of Health Guide for the Care and Use of Laboratory Animals and approved by the National Institute on Drug Abuse and National Institute of Mental Health Intramural Research Program Animal Care and Use Committees.

#### Operant alcohol self-administration and alcohol vapor exposure

Rats were trained to lever press for access to alcohol or water in standard operant chambers (Med Associates, St. Albans, VT, USA), as previously reported [32]. To habituate the rats to alcohol’s taste, they were given free access to alcohol (10%, w/v) and water for 1 day in their home cages. They were subsequently subjected to an overnight session in operant chambers with access to one lever (right lever) that delivered water (100 μl) with food freely available. After 1 day off, rats were subjected to a 2-h session followed by a 1-h session (the next day), with one lever that delivered alcohol (right lever). The subsequent sessions lasted 30 min, and two levers were available (left lever: water; right lever: alcohol). The operant sessions were conducted on a fixed ratio 1 schedule of reinforcement (i.e., each lever press resulted in fluid delivery). Upon stable levels of responding to alcohol, the rats were separated into two groups: (1) exposed to chronic, intermittent alcohol vapor to induce dependence (dependent rats); (2) exposed to air (nondependent rats). Cycles of alcohol intoxication and withdrawal occurred daily for 6 weeks. Over a 24-h period, the alcohol vapor was ON for 14 h (from 6 p.m. to 8 a.m.) consecutively, and operant alcohol self-administration (twice per week) occurred during the 10-h period without alcohol vapor between 6 and 8 h into withdrawal. In this model, rats exhibit reliable signs of alcohol dependence, including a negative emotional-like state and somatic symptoms during withdrawal (for review, see refs. [33, 34]). During vapor exposure, the target blood alcohol levels were 150–250 mg/dl that was maintained for at least 4 weeks.

#### PET study

The rats were removed from vapor around 6 a.m. and transported by car with temperature controlled (21 ± 2 °C) from the Baltimore to the Bethesda Campus in less than 2 h. Alcohol-dependent (*n* = 10; weight: 547 ± 40 g) and -nondependent rats (*n* = 10; weight: 573 ± 60 g) were scanned in pairs under anesthesia (2–5% isoflurane). One dependent rat was removed from the study due to radiotracer administration failure. Each rat was scanned between 11 a.m. and 4 p.m. (i.e., 5–10 h into withdrawal). A pair of rats (one dependent and one nondependent) was placed side by side in prone position in a small animal PET scanner (microPET Focus-220, Siemens). Following a 10-min transmission scan using Co-57 as the source, [^11^C]PBR28 (mean ID, 459 ± 151 μCi; A_m_ >0.34 Ci/μmol) was administrated intravenously. Data acquisition was started simultaneously, and continued for 60 min in list mode. Thirty minutes after the end of the [^11^C]PBR28 scan, a 30-min [^18^F]FDG (100–400 μCi) scan was obtained for anatomical coregistration with the [^11^C]PBR28 images.

#### Image analysis

The Wistar rat brain template [35] was used for coregistration and for defining ROIs (whole brain, gray matter, white matter, hippocampus, and thalamus). For each animal, their [^18^F]FDG brain images were used as template to coregister their [^11^C]PBR28 images using PMOD. The coregistration parameters were applied to the [^11^C]PBR28 images and used to generate time–activity curves for the ROIs.

## Results

### Human clinical characteristics

Table 1 provides a summary on demographics and clinical characteristics. AUD patients drank an average of 8.9 ± 4.9 alcoholic drinks per day in the 90 days prior to the study, with an average of 5.9 ± 1.5 drinking days per week. HC drank 0.1 ± 0.2 drinks per day on an average of 0.4 ± 0.7 days per week. AUD patients had higher trait anxiety scores than HC (*p* = 0.001).

HC had higher IQ scores compared to AUD (*t* = 2.3, *p* = 0.03) and there were ten smokers in the AUD group, but none in the HC group (*χ*^2^ = 12.4, *p* < 0.0001; see Table 1). Since smoking is associated with lower TSPO binding in brain [36], we also assessed whether there were differences in the percentage of smokers between *TSPO* genotypes. The high-binder group tended to have a larger percentage of smokers (7 smokers and 15 nonsmokers) than the middle-binder group (3 smokers and 11 nonsmokers) for the AUD and HC pooled together but the difference was not significant (*χ*^2^ = 0.46, *p* = 0.50). Similarly, for the AUD group, the high-binder group tended to have a higher percentage of smokers (seven smokers and four nonsmokers) than the middle-binder group (three smokers and five nonsmokers), which was also not significant (*χ*^2^ = 1.27, *p* = 0.26).

C-reactive protein and cholesterol levels were within the normal range in both groups and there were no group differences (see Supplementary Table 1). ACTH, cortisol, and liver AST were higher in AUD compared to HC (all *p* > 0.05).

#### Human PET [^11^C]PBR28

**Fig. 1:**
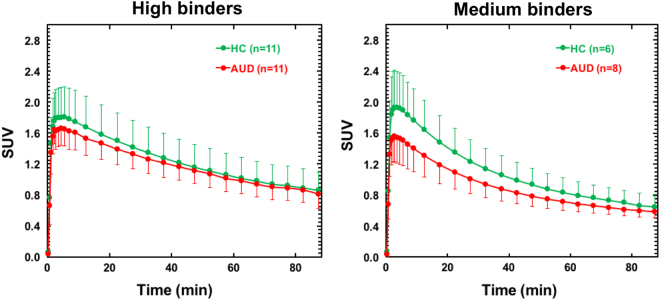
Average [^11^C]PBR28 whole brain time–activity curves in AUD and HC for high- and medium-affinity binders. Bars represent standard deviation. SUV standard uptake value

Group mean images and time–activity curves for [^11^C]PBR28 SUV in the whole brain are shown in Fig. 1.

**Fig. 2:**
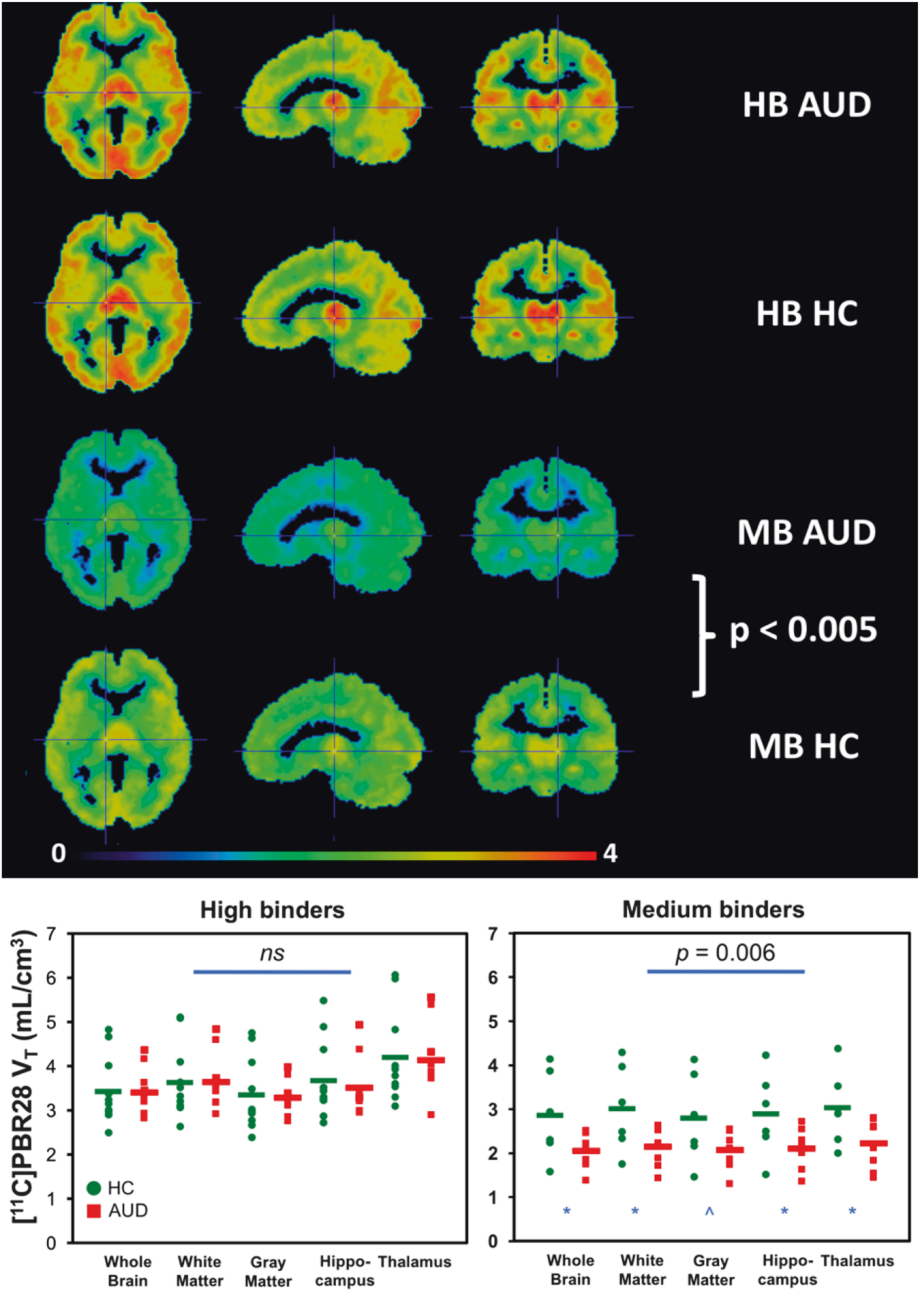
[^11^C]PBR28 *V*_T_ is lower in AUD versus HC in medium-affinity binders for whole brain, white matter, hippocampus, and thalamus; and at trend level for gray matter

There was a significant main effect of *TSPO* genotype rs6971 on [^11^C]PBR28 *V*_T_ (Θ = 0.97, *F*_5,27_ = 5.2, *p* = 0.002, *η*^*2*^ = 0.49); high-affinity binders had higher *V*_T_ in all ROIs (all *p* < 0.0001). There was no main group effect (Θ = 0.18 F_5,27_ = 1.0, *p* = 0.46, *η*^*2*^ = 0.15), no interaction effect of rs6971 × group (Θ = 0.33, *F*_5,27_ = 1.8, *p* = 0.16, *η*^*2*^ = 0.25), and no effect of age (Θ = 0.30, F_5,27_ = 1.6, *p* = 0.19, *η*^*2*^ = 0.23) on [^11^C]PBR28 *V*_T_. However, when separating the analyses by genotype, there was a significant group effect in medium-affinity binders only (Θ = 6.36, F_5,7_ = 8.9, *p* = 0.006, *η*^*2*^ = 0.86) with univariate tests showing lower [^11^C]PBR28 *V*_T_ in AUD compared to HC in whole brain (*F*_14_ = 6.5, *p* = 0.027), gray matter (*F*_14_ = 6.9, *p* = 0.023), white matter (*F*_14_ = 4.8, *p* = 0.05), hippocampus (*F*_14_ = 6.4, *p* = 0.028), and thalamus (*F*_14_ = 6.7, *p* = 0.025) (Fig. 2).

#### Correlations with cholesterol, cortisol, and ACTH

**Fig. 3:**
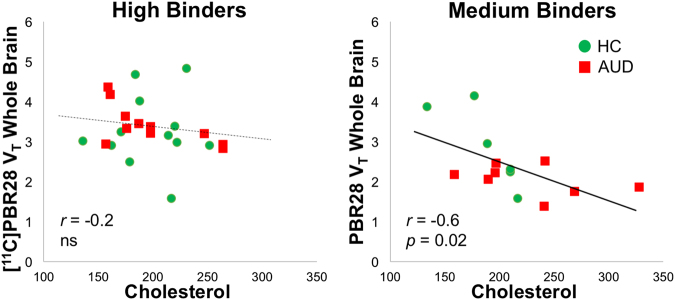
Cholesterol correlated negatively with whole brain [^11^C]PBR28 *V*_T_ in groups pooled together corrected for genotype (cholesterol: *r*_33_ = −0.4, *p* = 0.02); largely driven by a negative correlation in AUD participants (*r*_16_ = −0.5, *p* = 0.02), and by a significant correlation in medium-affinity binders (*r*_14_ = −0.6, *p* = 0.02)

Cholesterol in plasma correlated negatively with whole brain [^11^C]PBR28 *V*_T_ in groups pooled together (*r*_33_ = −0.39, *p* = 0.02; corrected for rs6971), largely driven by a negative correlation in AUD patients (*r*_16_ = −0.53, *p* = 0.02) but not in controls (*r*_14_ = −0.46, *p* = 0.45, ns). The correlation was signifcaint in medium-affinity binders only (*r*_14_ = −0.60, *p* = 0.02) (Fig. 3). (Note that in medium-binder HC the correlation was also significant *r*_6_ = −0.85, *p* = 0.03, but not in HC high binders).

Cortisol in plasma showed a trend of a negative correlation with whole brain [^11^C]PBR28 *V*_T_ in groups pooled together (*r*_30_ = −0.34, *p* = 0.06; corrected for rs6971), but there were no associations between cortisol and [^11^C]PBR28 binding in AUD or HC seperately. Separate analyses by genotype revealed that in medium-affinity binders whole brain [^11^C]PBR28 *V*_T_ was negatively associated with cortisol levels (*r*_14_ = −0.74, *p* = 0.002); however, since for medium binders both *V*_T_ and cortisol were lower in AUD than controls, this correlation might have been driven by the group differences. The correlation between ACTH levels and brain [^11^C]PBR28 *V*_T_ were not significant either for the pooled or the separate analyses.

Cortisol and cholesterol plasma levels were positively correlated at trend level (*r* = 0.31, *p* = 0.08). Supplementary Tables 2 and 3 provide zero-order correlations and linear regression models on the effects of AUD diagnosis, rs6971, age, BMI, smoking status, cholesterol, cortisol, and ACTH with brain [^11^C]PBR28 *V*_T_, showing that in addition to rs6971, cholesterol (and cortisol at trend level) was an independent predictor of [^11^C]PBR28 *V*_T_.

### PET [^11^C]PBR28 in rodents

Alcohol-dependent rats exhibited escalated responding for alcohol compared with nondependent rats (dependent: 55.6 ± 2.4 alcohol deliveries in 30 min; nondependent: 35.4 ± 3.3 alcohol deliveries in 30 min; *p* < 0.05). Note that the dependent rats received more than 4 weeks of blood alcohol levels ranging from 150 to 250 mg/dl during the 14 h of alcohol vapor exposure every day. The dependent and nondependent rats were submitted to 30-min oral self-administration sessions twice a week for 6 weeks. During the self-administration sessions, the average blood alcohol levels of nondependent rats were estimated to be less than 50 mg/dl, whereas the average blood alcohol levels of dependent rats were estimated to be around 100 mg/dl.

There were no significant differences in the time–activity curves for [^11^C]PBR28 SUV in the brain of alcohol-dependent and -nondependent rats (Supplementary Figure 2).

**Fig. 4:**
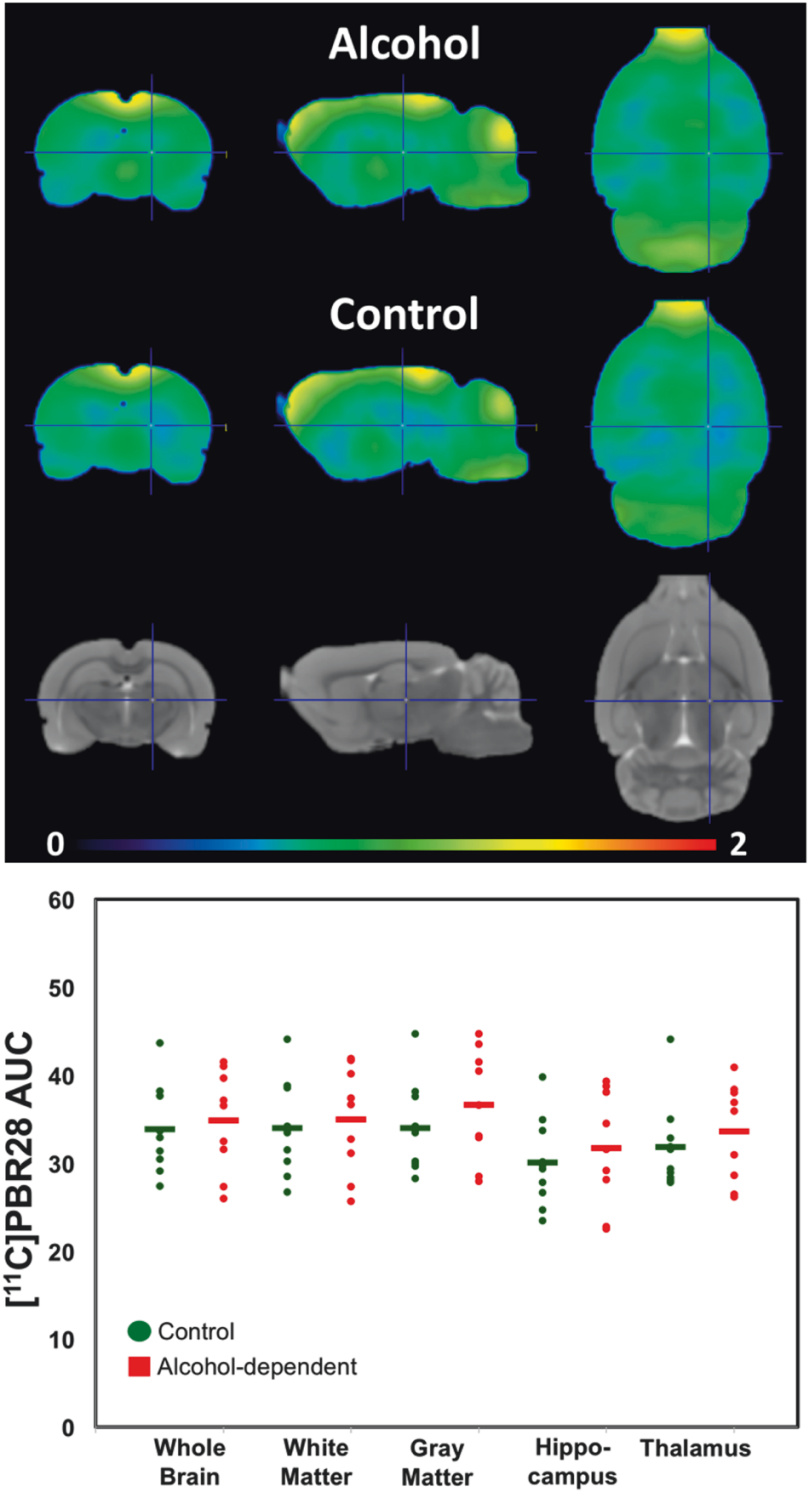
No differences in [^11^C]PBR28 binding were found between alcohol-dependent versus -nondependent rats for whole brain, cortical gray matter, hippocampus, or thalamus

Multivariate analyses on the comparison of the SUV measure in all ROIs showed a trend effect for higher values in alcohol-dependent than -nondependent rats (Θ = 0.97, *F*_5,13_ = 2.5, *p* = 0.083, *η*^*2*^ = 0.49). However, the univariate analyses showed no differences between alcohol-dependent and -nondependent rats in whole brain, gray matter, white matter, hippocampus, or thalamus (*p* > 0.3; Fig. 4).

## Discussion

We found no AUD group differences on [^11^C]PBR28 binding in our clinical study and no group differences in [^11^C]PBR28 uptake in the brain of alcohol-dependent versus -nondependent rats. However, when separately analyzing by *TSPO* genotype, we found lower [^11^C]PBR28 binding in the brain of AUD patients than in HC for medium-affinity binders only. We also corroborated our hypothesis of an inverse association between [^11^C]PBR28 binding and plasma cholesterol levels that was significant in groups pooled together, due to significance in medium-affinity binders.

These findings contrast with those from previous preclinical in vitro autoradiography studies that showed higher binding of the TSPO tracer [^11^C]PK-11195 and of the TSPO ligand [^11^C]DAA1106 in rats 3–7 days after intrastriatal injection on alcohol [37]; and those from postmortem brain studies that showed increases in the expression of genes involved in neuroinflammation in alcoholics compared to controls [17]. Indeed, when we initially designed this study, we had expected to find elevated [^11^C]PBR28 binding in the brain of AUD compared to HC, consistent with chronic alcohol-induced neuroinflammation. However, after two recent clinical PET studies showed lower [^11^C]PBR28 binding in AUD compared to controls, which suggested that neuroinflammation in AUD might not be associated with microglial activation [18, 19], we changed our hypothesis to predict that we would replicate [^11^C]PBR28 decreases in the brain of AUD and in rodents chronically exposed to alcohol.

Although we found no group differences between AUD and HC in [^11^C]PBR28 brain binding when analyses were pooled across genotypes, separate analyses by genotype revealed lower [^11^C]PBR28 binding in AUD in medium-affinity binders but no differences in high-affinity binders. Furthermore, we did not observe differences in radiotracer uptake between alcohol-dependent and -nondependent rats. It is important to mention that both human high-affinity binders and all rats have an alanine in their TSPO-binding site (these groups show similar results), whereas human medium-affinity binders express equal amounts of TSPO-binding sites that have either an alanine or a threonine residue. These findings inspire multiple interpretations. One explanation for decreased [^11^C]PBR28 binding in AUD proposed previously [18, 19] could be a loss of microglia or astrocytes in AUD. A previous postmortem study found lower microglia and astrocytes in AUD in various brain regions, including the hippocampus [38]. However, this interpretation would not explain why we did not find group differences in brain [^11^C]PBR28 binding in the high-affinity binders nor in our preclinical model of alcohol dependence. Second, TSPO expression may be decreased due to potential loss of mitochondrial density with chronic alcohol exposure [19] as has been reported to occur in preclinical models of alcoholism [39]. Furthermore, suppressed neurogenesis in AUD may contribute to the group differences as TSPO is also expressed by neural stem cells [40]. Although this again would not explain the lack of an effect in the high-affinity binders nor in our preclinical model.

Finally, our most plausible explanation is that an endogenous TSPO ligand is expressed more in AUD participants than controls, competing with [^11^C]PBR28 for the TSPO-binding site. Note that the amino acid substitution of the TSPO polymorphism is located in the cholesterol-binding domain of TSPO [23], which could result in different sensitivity to competition by an endogenous ligand as a function of genotype and might explain why AUD differences were only observed in middle-affinity binders as was the association with cholesterol. Competition with an endogenous TSPO ligand would explain the discrepancy between current and prior in vivo findings and prior in vitro autoradiography findings in rat models of alcohol abuse; all of which found increased TSPO expression in hippocampus compared to non-drinking rats [12, 37]. Moreover, in follow-up autoradiographic studies in the brain of the rats that were scanned with PET and [^11^C]PBR28, revealed a significant increase in [^3^H]PBR28 in the hippocampus of the alcohol-dependent rats in whom the PET studies showed no differences (Tyler et al., manuscript in preparation). Difference in the findings with alcohol exposure between in vivo (showing lower TSPO ligand binding) and in vitro (showing increases in TSPO ligand binding) could be explained by increases in an endogenous TSPO ligand with chronic alcohol, which would compete with radiotracer binding in vivo but not in vitro. Supporting this interpretation are preclinical findings that have reported increases in the brain concentration of the diazepam-binding inhibitor (DBI), which is an endogenous TSPO ligand [41] and its mRNA with chronic alcohol exposure [42, 43] as well as clinical studies showing increases in DBI in the cerebrospinal fluid of alcoholics compared to that of controls [44, 45].

We found the first evidence of an inverse correlation between cholesterol and [^11^C]PBR28 *V*_T_ in all groups pooled together (HC + AUD), which was driven by a significant correlation in the AUD group, and medium-affinity binders (Supplementary Table 2). Given that TSPO has a cholesterol-binding domain that affects the conformation of TSPO if cholesterol is bound [20], endogenous concentrations of cholesterol in blood and brain could affect binding of [^11^C]PBR28 to TSPO, potentially influencing our results. Moreover, because the *TSPO* rs6971 polymorphism leads to an amino acid substitution located at the site of the TSPO cholesterol-binding domain [23]. This could lead to differential sensitivity to cholesterol’s effects on TSPO and could explain why the inverse association with cholesterol was observed in medium-affinity binders but not in high binders. Although we did not see group differences in AUD and HC in cholesterol levels, cholesterol levels could have contributed to the lower [^11^C]PBR28 binding observed in AUD. Indeed, abnormalities in cholesterol levels have been associated with cognitive impairments in alcoholism [8]. Moreover, in our multiple regression model, we see cholesterol as independent predictor of [^11^C]PBR28 binding along with *TSPO* genotype rs6971. Thus, future studies should take cholesterol levels into account when measuring [^11^C]PBR28 binding in the brain.

We also observed a significant inverse association between [^11^C]PBR28 binding in brain and levels of cortisol in plasma of medium-affinity binders only. This is consistent with findings that *TSPO* deletion in rodents [46] and its corresponding rs6971 polymorphism in humans has recently been associated with diurnal variation (morning versus evening in cortisol levels in saliva in patients with bipolar disorder and AUD [47]. In our sample, all blood draws for plasma cortisol, cholesterol, and ACTH analyses were drawn in the morning, thus investigating effects of rs6971 on diurnal variance in cortisol was not possible; and tracer injection time (ranging from 10 a.m. to 2:30 p.m.) did not correlate with whole brain [^11^C]PBR28 binding in groups pooled together. *TSPO* rs6971 has furthermore been shown to alter ACTH-induced plasma corticosteroid concentrations [23]. ACTH levels were not correlated with [^11^C]PBR28 binding in the brain. In flies, TSPO mRNA expression was reported to be significantly lower in females versus males [13]. Nevertheless, in our studies, we did not find evidence for gender differences in [^11^C]PBR28 binding in brain. Benzodiazepines bind to TSPO [48], but we did not see differences in [^11^C]PBR28 binding in our AUD patients who were given benzodiazepines to control their withdrawal symptoms (i.e., three AUD patients used benzodiazepines 2 days before the study and two on the study day) compared to those who did not receive them.

Limitations of the study include the small sample size, which might have limited our ability to detect significant differences in high-affinity binders. However, despite the small sample, we did find lower [^11^C]PBR28 binding in medium-affinity binders AUD versus HC participants, and showed an inverse association of [^11^C]PBR28 with plasma cholesterol and cortisol levels that was also restricted to the medium-affinity binders. This contrasts with findings from Kalk et al. [19] who reported that group differences were more pronounced in high- rather than medium-affinity binders (although this finding is based on only three high-affinity binder AUD patients), whereas Hillmer et al. [18] provide no further analyses on medium-affinity versus high-affinity binders. Because [^11^C]PBR28 has lower-specific binding to TSPO, we speculate that medium-affinity binders may be more sensitive to [^11^C]PBR28 binding competition with endogenous TSPO ligands, such as cholesterol, that might be influenced by chronic alcohol exposures [49]. Moreover, our groups were not matched for smoking status, which is relevant since human smokers were recently shown to have lower brain binding of the TSPO ligand [^11^C]DAA1106 than nonsmoking controls in vivo [36]. However, here we showed no effect of smoking on [^11^C]PBR28 binding (Supplementary Tables 1 and 2), which could reflect lower number of cigarettes smoked per day in our participants (11 ± 8) versus that of Brody et al. (14 ± 4 SD) and or differences in radiotracers or other sample characteristics.

In summary, we found evidence for lower [^11^C]PBR28 binding in the brain of AUD patients with a medium-affinity binding genotype and no differences in an animal model of alcohol dependence. This could reflect disrupted microglia activation during alcoholism and/or competition with an alcoholism-associated increase of endogenous TSPO ligand(s) observed in those with a medium-affinity binder genotype.

## Electronic supplementary material

Supplemental Material
