# Supplementary material for: Influence of alcoholism and cholesterol on TSPO binding in brain: PET [11C]PBR28 studies in humans and rodents
Source: Neuropsychopharmacology. 2018 May 3;43(9):1832–9. doi: 10.1038/s41386-018-0085-x (PMC6046047; doi:10.1038/s41386-018-0085-x)
Supplement: Supplementary file 1 — Supplemental Material [file 41386_2018_85_MOESM1_ESM.doc]

**Supplementary Material to: Influence of alcoholism and cholesterol on TSPO binding in brain: PET [^11^C]PBR28 studies in humans and rodents**

Sung Won Kim^1*^, Corinde E. Wiers^1*^, Ryan Tyler^1^, Ehsan Shokri-Kojori^1^, Yeon Joo Jang^1^, Amna Zehra^1^, Clara Freeman^1^, Veronica Ramirez^1^, Elsa Lindgren^1^, Gregg Miller^1^, Elizabeth Cabrera^1^, Tyler Stodden^1^, Min Guo^1^, Şükrü B. Demiral^1^, Nancy Diazgranados^1^, Luke Park^2^, Jeih-San Liow^2^, Victor Pike^2^, Cheryl Morse^2^, Leandro F. Vendruscolo^3^, Robert B. Innis^2^, George F. Koob^3^, Dardo Tomasi^1^, Gene-Jack Wang^1^, Nora D. Volkow^1^

**Supplemental Material 1. Gray Matter Volume differences in AUD *vs.* HC.**

Data processing and analysis were performed with the Statistical Parametric Mapping 8

(SPM8) software package (www.fil.ion.ucl.ac.uk/spm; Welcome Department of Imaging

Neuroscience), using the VBM toolbox for SPM, version 8 (dbm.neuro.uni-jena.de/vbm8). After normalization to the stereotaxic standard space as defined by the template provided by the International Consortium of Brain Mapping (ICBM, http://www.loni.usc.edu/ICBM/), T1-weighted MPRAGE images were segmented into gray matter, white matter and cerebrospinal fluid, using diffeomorphic anatomical registration through exponential lie algebra (DARTEL; Ashburner, 2007). Gray matter tissue probability maps (TPMs) were then spatially smoothed with an isotropic Gaussian kernel with 10 mm full-width at half-maximum.

The smoothed gray matter TPMs were statistically analyzed by means of a two-sample t-test with group (AD, HC) as the between-subjects factor. No results passed an a-priori statistical threshold of p<0.05, family wise error (FWE) corrected, and a minimal cluster size of at least 50 adjacent voxels. At a more liberal threshold of *p*<0.001 uncorrected, *k*>50. However, AUD patients showed decreases in GMV in left superior temporal gyrus (coordinates MNI [*x, y, z*]= [-52, -54, 24], *t*=4.2, *k*=129, *p*<0.001), right middle temporal gyrus ([46, -6, -32], *t*=4.1, *k*=50, *p*<0.001).


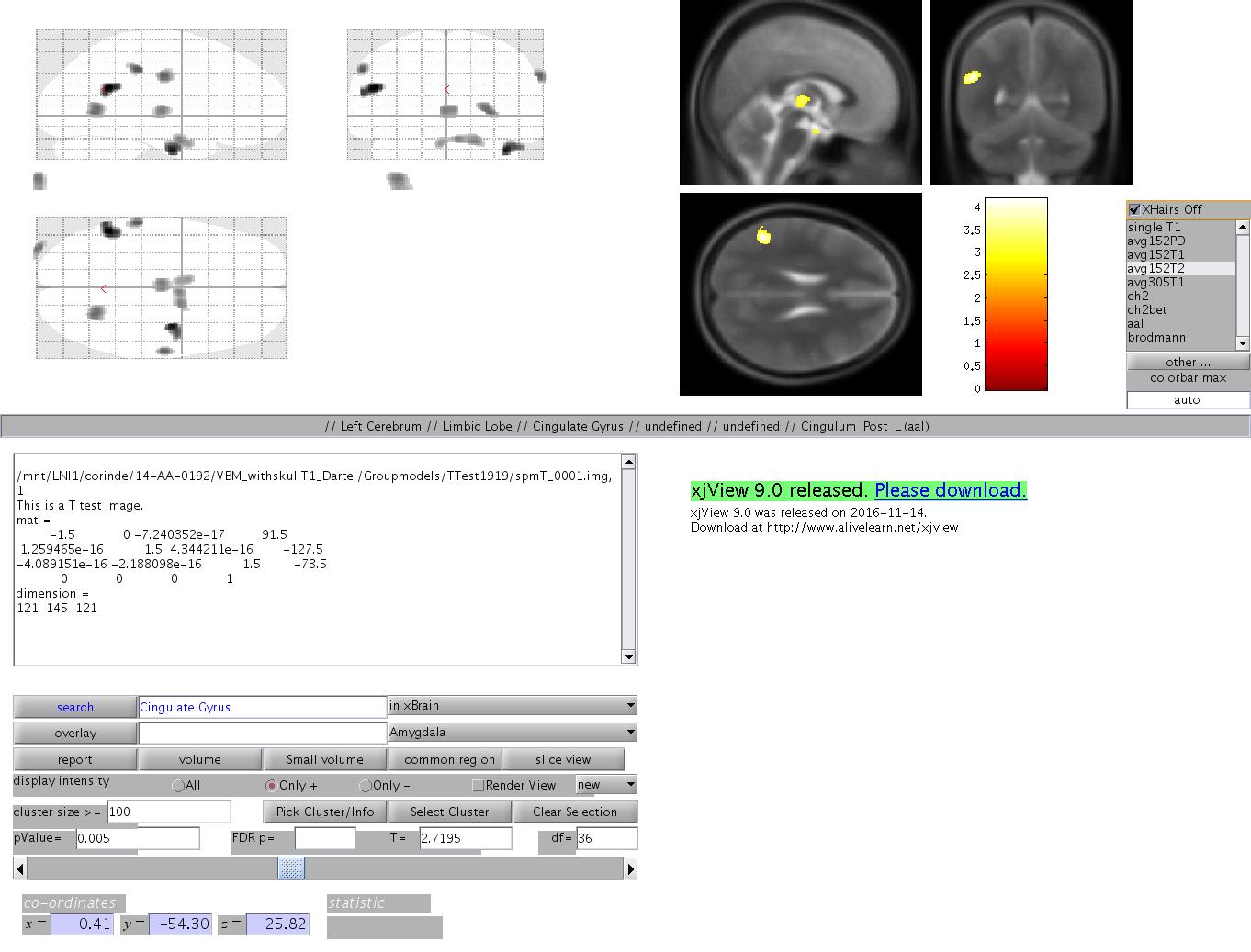


***t***

**Supplemental Figure 1.** AUD patients showed decreases in GMV in left superior temporal gyrus (coordinates MNI [x, y, z]= [-52, -54, 24], *t*=4.2, k=129, *p*<0.001), right middle temporal gyrus ([46, -6, -32], t=4.1, k=50, *p*<0.001)

**Supplementary Table 1. Zero-order correlations between AUD diagnosis, rs6971, age, BMI, smoking status, cholesterol, ACTH and whole brain [^11^C]PBR28 V_T_ .**

|  | AUD dx | rs6971 | age | BMI | Smoke  status | Cholesterol | Cortisol | ACTH | WB PBR28 |
| --- | --- | --- | --- | --- | --- | --- | --- | --- | --- |
| AUD dx | 1 |  |  |  |  |  |  |  |  |
| rs6971 | -.070 | 1 |  |  |  |  |  |  |  |
| age | .005 | -.117 | 1 |  |  |  |  |  |  |
| BMI | -.163 | -.133 | **-.291^** | 1 |  |  |  |  |  |
| Smoke | **.587^**^** | .113 | .167 | -.158 | 1 |  |  |  |  |
| Cholesterol^a^ | .208 | -.165 | .119 | .077 | .026 | 1 |  |  |  |
| Cortisol^b^ | **.413^*^** | -.105 | .137 | **-.292^** | **.309^** | **.309^** | 1 |  |  |
| ACTH | **.383^*^** | -.228 | -.226 | -.118 | .291 | **.393^*^** | .275 | 1 |  |
| WB PBR28 | -.228 | **.592** | -.259 | -.124 | -.088 | **-.406^*^** | **-.332**^ | -.273 | 1 |

^**^*p*<0.01 ^*^*p*<0.05 ^*p*<0.1, ^a^*n*=33 ^b^*n*=32

**Supplementary Table 2.** Summary of regression analyses for AUD diagnosis (dx) and covariates predicting whole brain [^11^C]PBR28 V_T_

|  | [^11^C]PBR28 V_T_ | | |
| --- | --- | --- | --- |
| *Variable* | *B* | *SE B* | *β* |
| AUD dx | 0.07 | 0.28 | 0.04 |
| rs6971 | 0.67 | 0.23 | **0.40**** |
| Age | -0.02 | 0.01 | -0.31 |
| BMI | -0.03 | 0.03 | -0.17 |
| Smoke | -0.09 | 0.29 | -0.05 |
| Cholesterol | -0.01 | 0.003 | **-0.35*** |
| Cortisol  ACTH | -0.05  -0.002 | 0.03  0.006 | **-0.27^**  -0.06 |
| *R^2^* | 0.68 | | |
| *F* | **5.84**** | | |

***p*<0.01 **p*<0.05 ^*p*<0.1

**
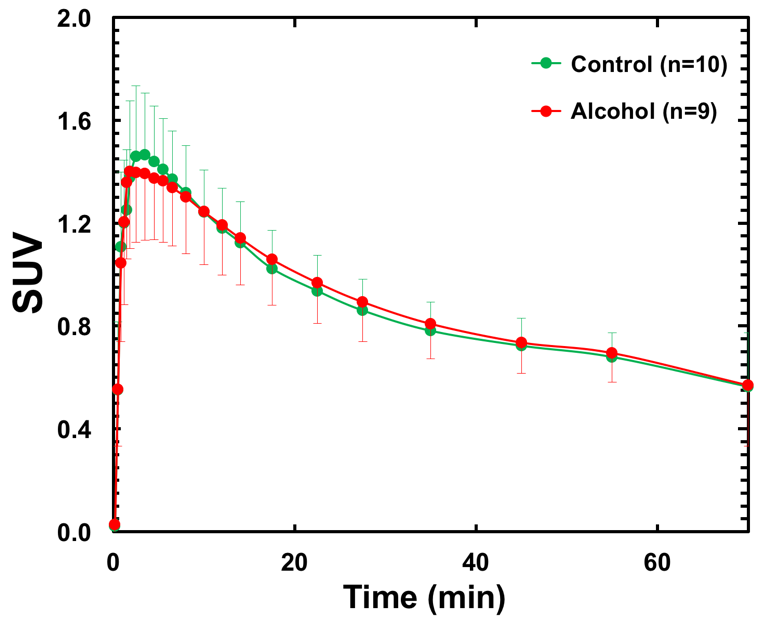
**

**Supplementary Figure 2.** [^11^C]PBR28 whole brain time activity curves in alcohol dependent (*n*=9) and nondependent control (*n*=10) rats. Bars represent standard deviation. SUV, standard uptake value.
